# Effect of Ultrasonic Treatment of Dispersed Carbon Nanocomposite Media on the Formation, Electrical Conductivity, and Degradation of a Hydrogel for Metallic Stimulation Electrodes

**DOI:** 10.3390/gels11121004

**Published:** 2025-12-12

**Authors:** Mikhail Savelyev, Artem Kuksin, Denis Murashko, Ekaterina Otsupko, Victoria Suchkova, Kristina Efremova, Pavel Vasilevsky, Ulyana Kurilova, Sergey Selishchev, Alexander Gerasimenko

**Affiliations:** 1 Institute of Biomedical Systems, National Research University of Electronic Technology, 124498 Zelenograd, Russia; 8140241@edu.miet.ru (A.K.); 8140918@edu.miet.ru (D.M.); 8191388@edu.miet.ru (E.O.); suchkova_v_v@staff.sechenov.ru (V.S.); popovich_k@staff.sechenov.ru (K.E.); u130020@edu.miet.ru (P.V.); kurilova_u_e@staff.sechenov.ru (U.K.); selishchev@bms.zone (S.S.); gerasimenko@bms.zone (A.G.); 2 Institute for Bionic Technologies and Engineering, I. M. Sechenov First Moscow State Medical University, 119991 Moscow, Russia

**Keywords:** hydrogel, photoresist, temperature, dynamic light scattering, nonlinear absorption cross-section, carbon nanotubes, reduced graphene oxide, biocompatibility, neurointerface

## Abstract

This study investigates the impact of ultrasonic treatment on the deagglomeration of aggregates of single-walled carbon nanotubes (SWCNTs) and reduced graphene oxide (rGO). The aim of the research is to enhance the electrical conductivity of a biopolymer hydrogel designed for coating metallic neurostimulation electrodes. Biocompatible coating materials are essential for the safe long-term function of implants within the body, enabling the transmission of nerve impulses to external devices for signal conversion and neurostimulation. Dynamic light scattering (DLS) was employed to monitor the dispersion state, in conjunction with measurements of specific electrical conductivity. The mass loss and swelling capacity were evaluated over an 80-day period to account for the effects of degradation during in vitro studies. Samples of flexible–elastic hydrogels for electrodes with complex geometry were formed by the photopolymerization of a photopolymerizable medium, similar to a photoresist. Analysis of the dependence of temperature and normalized optical transmittance on the duration of laser photopolymerization made it possible to determine the optimal polymerization temperature for the photopolymerizable medium as −28 °C. This temperature regime ensures maximum reproducibility of hydrogel formation and eliminates the presence of unpolymerized areas. The article presents a biopolymer hydrogel with SWCNTs and rGO nanoparticles in a 1:1 ratio. It was found that sufficient specific electrical conductivity is achieved using SWCNTs with a characteristic hydrodynamic radius of R = 490 nm and rGO with R = 210 nm (sample Col/BSA/CS/Eosin Y/SWCNTs (490 nm)/rGO **4**). The photopolymerized hydrogel **4** demonstrated sufficient biocompatibility, exceeding the control sample by 16%. According to the results of in vitro studies over 80 days, this sample exhibited moderate degradation of 45% while retaining its swelling ability. The swelling degree decreased by 50% compared to the initial value of 170%. The presented hydrogel 4 is a promising coating material for implantable metallic neurostimulation electrodes, enhancing their stability in the physiological environment.

## 1. Introduction

Acceleration and activation of ionic processes in cellular activity can be achieved using electrical stimulation [1,2]. This initiates the transmission of impulses directly by cells through internal pathways with the activation of ion channels. This is necessary for stimulating biological tissue for therapeutic purposes, for restoring nerve conduction [3,4] and alleviating chronic pain in patients with spinal cord injuries [5], and for muscle stimulation [6]. To achieve this effect, electrodes made of conductive and biocompatible materials are being developed [2,7,8]. Their purpose is to deliver high-current and high-energy signals over long periods of time to block pain [2,9]. Unintentional blockade of afferent nerves can cause undesirable reactions in the central nervous system (CNS) and worsen the patient’s condition; therefore, it is crucial that the electrode array provides targeted and localized delivery of modulating signals over an extended period. The key task is to create a stable electrode–neuron interface that maintains the ability to transmit adequate and localized signals over a chronic time interval [10].

Recent studies have shown that charge-unbalanced direct current (DC) signals [11,12] or prolonged electrical impulses can reversibly suppress neuronal activity [13,14]. This demonstrates the potential of such signals for reversible blockade of peripheral nerves while preserving somatosensory and motor conduction, opening possibilities for targeted pain therapy [9,11,15].

The expansion of indications for spinal cord stimulators and progress in spinal cord stimulation (SCS) equipment have accelerated the development of therapeutic programming methods [16]. SCS signals are characterized by frequency, pulse width, amplitude, and pulse shape [5,17,18]. The prototypical form is traditional (tonic) stimulation—relatively low frequency (40–80 Hz), large pulse width (200–500 µs), and high amplitude (3.5–8.5 mA)—which leads to an increase in charge per pulse and the generation of action potentials in the stimulation area. Activation of large A-beta fibers (Erlanger-Gasser classification) causes a sensation of paresthesia—a characteristic feature of tonic SCS.

Classical studies have demonstrated that effective analgesia with traditional SCS requires the paresthesia area to overlap with the pain zone [19,20]. Achieving this overlap involves adjusting the amplitude and position of the electrodes through intraoperative mapping and postoperative programming. As tonic stimulation is applied over long periods and is more extensively studied, numerous investigations have focused on its efficacy and mechanisms of action. One primary mechanism is believed to involve the activation of A-beta fibers, followed by the activation of inhibitory interneurons in the dorsal horn of the spinal cord, which attenuates the transmission of pain signals. Additionally, orthodromic activation may engage supraspinal pathways, thereby enhancing analgesia [5,17,18].

Metal electrodes are limited in their ability to deliver stable signals over long periods of time in the body’s physiological environment due to the encapsulation effect [21,22]. Furthermore, for their long-term practical application, it is necessary to ensure breathability (a stagnant water layer between the electrode and the skin leads to signal drift and even causes allergies and skin inflammation), address the issue of mechanical mismatch with soft nervous tissue (metals, as a rule, cannot deform by more than 5% without failure, which affects their functionality and long-term use), and ensure a tight fit considering the mobility of nervous tissues (the material must be able to stretch while maintaining its shape without breaking adhesion) [5,18,23]. To solve these problems, the present work employs an approach involving the use of conductive hydrogels as a coating for electrodes [24]. Their application enhances the efficiency and operational stability of bionic devices designed to substitute or augment the functions of sensory neurons [25,26].

The use of hydrogels as coatings with sufficient electrical conductivity helps reduce the corrosion of metal electrodes due to the possibility of using lower currents [1,2,7]. To ensure biocompatibility, the use of collagen (Col), bovine serum albumin (BSA), and chitosan (CS) is being considered, although these materials themselves lack the necessary electrical conductivity [24,27]. To address this challenge, the use of single-walled carbon nanotubes (SWCNTs) is proposed. However, their application is limited by insufficient knowledge of their toxic effects. Therefore, it is important to have preparation methods that allow for the fullest realization of their potential [28] and, consequently, their use in the minimum possible quantity. Another important consideration is the availability of biocompatible coatings, such as BSA or CS, as used in [24,29,30]. Furthermore, other allotropic forms of carbon can exhibit lower toxicity compared to SWCNTs [31], which may allow the use of larger quantities of such particles in hydrogels without a significant reduction in biocompatibility. Previously, our work described the possibility of increasing current emission using a precise combination of graphene with SWCNTs under laser radiation, resulting in the formation of a recombinant graphene/SWCNT structure [32].

Obtaining a highly conductive hydrogel while using a minimal amount of SWCNTs is complicated by the nanotubes’ tendency to agglomerate. The hydrophobic surface of the nanoparticles and van der Waals interactions lead to their sticking and the formation of large SWCNT tangles in an aqueous environment. Consequently, agglomerates isolated within the dielectric matrix cannot form a percolating conductive network [33]. Ultrasonic treatment is a common method for disrupting carbon nanoparticle agglomerates through cavitation. Using low power and varying the processing time enables targeted improvement of dispersion homogeneity without damaging or shortening the SWCNTs [34].

For this study, we employed SWCNTs whose bundle separation we monitored using the dynamic light scattering (DLS) method to obtain a homogeneous dispersed medium approaching the individual distribution of SWCNTs. We also incorporated reduced graphene oxide (rGO) into this dispersion. Controlling the size of SWCNT agglomerates and creating hybridized nanoparticles (graphene/SWCNTs) aimed to develop a strategy for enhancing hydrogel conductivity. We assessed the specific electrical conductivity of the fabricated Col/BSA/CS/Eosin Y/SWCNTs/rGO hydrogel.

We formed the hydrogel by polymerizing a photopolymerizable dispersion that functioned similarly to a negative photoresist.

Eosin Y served as the photoinitiator due to its ability to provide sufficient absorption at the laser wavelength of 1070 nm [24]. Laser irradiation in the presence of the Eosin Y photoinitiator induced free-radical cross-linking of the biopolymers, forming a three-dimensional covalent hydrogel network. Successful photopolymerization requires consideration of optical characteristics and temperature control to prevent irreversible denaturation of sensitive protein components [35,36,37]. The photopolymerization temperature also acts as a key indicator of the liquid-to-gel phase transition, necessary for precise microfabrication control.

Thus, this work investigates the correlation between the hydrodynamic radius of SWCNT agglomerates and the optical, thermal, and electrical characteristics of the hydrogel. The practical significance of the work lies in developing a methodology for controlling the material’s electrical conductivity, as well as a photopolymerization technology that ensures high reproducibility of complex microstructures. This article aims to develop a new generation of biocompatible coating materials for electrodes, capable of providing stable and long-term contact with nervous tissue, thereby expanding the possibilities of neurorehabilitation.

## 2. Results and Discussion

### 2.1. Dynamic Light Scattering Study

**Figure 1:**
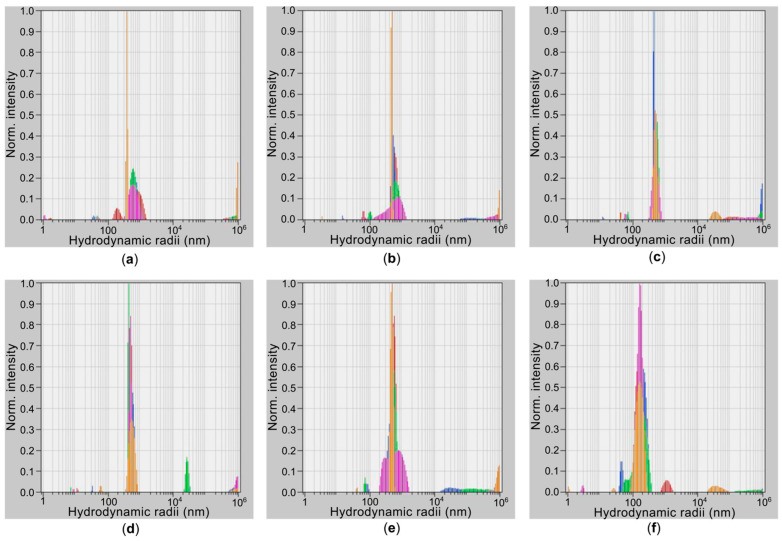
Results of DLS studies for dispersions with carbon nanoparticles after ultrasonic treatment with a power of 210 W: (**a**) SWCNT 20 min; (**b**) SWCNT 40 min; (**c**) SWCNT 60 min; (**d**) SWCNT 80 min; (**e**) SWCNT 100 min; (**f**) rGO 80 min. Hydrodynamic radius values are presented on a logarithmic scale.

Figure 1 shows results on hydrodynamic radius for SWCNT and rGO dispersions obtained using dynamic light scattering (DLS). The ultrasonic treatment of the dispersions with an immersion homogenizer affected the distribution of nanotubes. Filter paper with a pore size of 3–4 μm was used to remove large tangles that can form under the action of van der Waals forces [38]. Table 1 shows the corresponding results. After 20 min of treatment (Figure 1a), the hydrodynamic radius for most SWCNTs in the dispersion is observed at a level of *R* = 800 nm.

**Figure 2:**
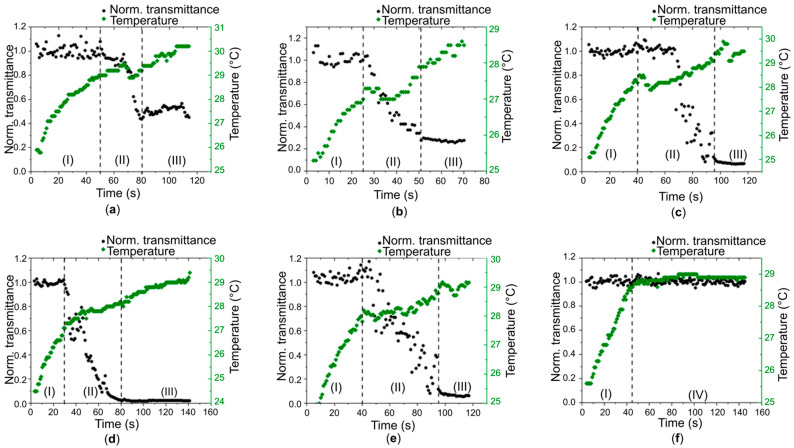
Dependences of temperature and normalized optical transmittance on the duration of laser photopolymerization of Col/BSA/CS/Eosin Y/SWCNTs/rGO dispersions: (**a**) **1**—SWCNTs (800 nm); (**b**) **2**—SWCNTs (600 nm); (**c**) **3**—SWCNTs (520 nm); (**d**) **4**—SWCNTs (490 nm); (**e**) **5**—SWCNTs (550 nm); (**f**) **4**—SWCNTs (490 nm) with a 3 mm offset from the lens focus. Area I is the temperature increase without any structural changes in the sample. Area II is a slow increase in temperature with a significant decrease in the normalized transmission coefficient. Area III is the achievement of a certain amount of heating without further temperature increase and the establishment of a stable balance between the energy parameters of the laser and the temperature at the site of exposure. Area IV is the absence of heating when setting a constant normalized transmission coefficient equal to 1.

In the case of studies of SWCNT dispersions, determining the actual sizes of nanotubes by the DLS method is difficult due to their shape and the ability to bend until they form tangles. The DLS method makes it possible to obtain the true sizes only for spherical particles, which are not SWCNTs and rGO. The use of a filter allowed us to remove tangles. At the same time the small diameter of the nanotubes and the small thinness of the graphene sheets allowed them to penetrate through the pores, which led to the appearance of peaks of a larger hydrodynamic radius in the graphs obtained by the DLS method. For the treatment times of 40 min (Figure 1b) and 60 min (Figure 1c), hydrodynamic radius decreased (*R* = 600 nm and *R* = 520 nm respectively). This indicates the separation of the initial bundles, which could be reflected in the decrease in the hydrodynamic radius. At the same time, during exposure to high-power ultrasound, shortening of SWCNTs can occur with the formation of many short segments [28,34], but no increase in the contribution to scattering from them was detected when determining the hydrodynamic radius. This effect could be facilitated by the moderate power value of 210 W during the treatment processing. For the treatment time of 80 min (Figure 1d), a state close to equilibrium occurs with the value of the hydrodynamic radius *R* = 490 nm, and further treatment (Figure 2e) does not lead to changes (*R* = 550 nm). During ultrasound exposure in the specified time range of up to 100 min, complete separation of the SWCNT bundles and their significant shortening does not occur. In the case of rGO, the minimum hydrodynamic radius value obtained under the same treatment conditions was approximately *R* = 210 nm at 80 min. At this value, sufficient dispersion and a virtual absence of adhered layers (agglomerates) were observed [34,39]. This duration of ultrasonic treatment was chosen as optimal for both types of carbon nanoparticles.

The prepared carbon nanoparticles were then used to prepare media intended to be used as a negative photoresist.

### 2.2. Temperature and Optical Density Control During Photopolymerization

Temperature has a significant effect when producing complex-shaped samples. To select its value for photopolymerization and reduce the number of unpolymerized areas, we studied the effect of laser radiation on the photocuring medium material. The following materials were used: Col 25 mg/mL, BSA 50 mg/mL, CS 100 mg/mL, Eosin Y 1 mg/mL, rGO 0.3 mg/mL, and SWCNTs 0.3 mg/mL. The different treatment time was used for SWCNTs (hydrodynamic radius *R* is shown in brackets): **1**—SWCNTs (800 nm); **2**—SWCNTs (600 nm); **3**—SWCNTs (520 nm); **4**—SWCNTs (490 nm); **5**—SWCNTs (550 nm). The results of the research are shown in Figure 2.

Using pulsed laser radiation with a power of approximately 190 mW and a repetition rate of 15 kHz, photopolymerization occurred over a prolonged period of up to 100 s. The laser energy parameters were selected to ensure sufficiently slow hydrogel formation, which eliminated the need for high-speed cameras and made it possible to use accessible measuring devices such as a pyrometer and thermal imager. Photopolymerization using the scanner system was performed at a higher radiation power, which made it possible to achieve photopolymerization in a shorter time. In the first seconds of exposure to radiation, sample **1** exhibited a temperature increase to 29 °C (Figure 2a) without any structural changes in the sample (area I). The rate of temperature increase then significantly decreased to less than 1 °C over 20 s, which was accompanied by a significant decrease in the normalized transmittance (area II). Upon completion of the hydrogel material formation, a constant value of the normalized transmittance was established, while the temperature continued to increase at approximately the same rate as in area I. Upon reaching a certain heating value, the further increase in temperature ceases and a stable equilibrium is established between the laser energy parameters and the temperature at the site of action (area III). A similar pattern was observed in other cases as well, with hydrogel formation occurring at temperatures of 27 °C (Figure 2b) and 28.5 °C (Figure 2c) for samples 2 and 3, respectively. A strong change in the normalized transmittance is observed, which may indicate the formation of a dense structure. In the case of sample 4 (Figure 2d), area II is located in the time interval from 35 to 80 s with a temperature of approximately 28 °C. Moreover, area III exhibits the lowest normalized transmittance of all the studied samples, indicating the most dense structure. For sample 5 (Figure 2e), the obtained temperature and normalized transmittance dependences on time are similar to those for sample 3. Figure 2f shows data for irradiating the sample out of focus (offset 3 mm). For this case, areas II and III were not detected; after heating by laser radiation (area I), area IV was observed, which is characterized by both the absence of heating and a constant normalized transmittance.

As a result of the analysis of the temperature and normalized optical transmittance dependences on the duration of laser photopolymerization, the optimal temperature of the liquid biopolymer dispersion was determined to ensure high reproducibility of the hydrogel characteristics obtained by photolithography. In the case of sample 5, the laser radiation power is also used most efficiently, as indicated by the value of the lowest normalized transmittance after the formation of the composite hydrogel. Under the action of pulsed laser radiation with a sufficient repetition rate, the hydrogel is formed with an increase in volume due to the addition of other SWCNTs and rGO [32,37], which form a framework, and the surface of the carbon nanoparticles is modified as a result of interaction with albumin [40] by analogy with a linear polymer [41].

### 2.3. Nonlinear Optical Properties of Dispersions for Hydrogel Creation

**Figure 3:**
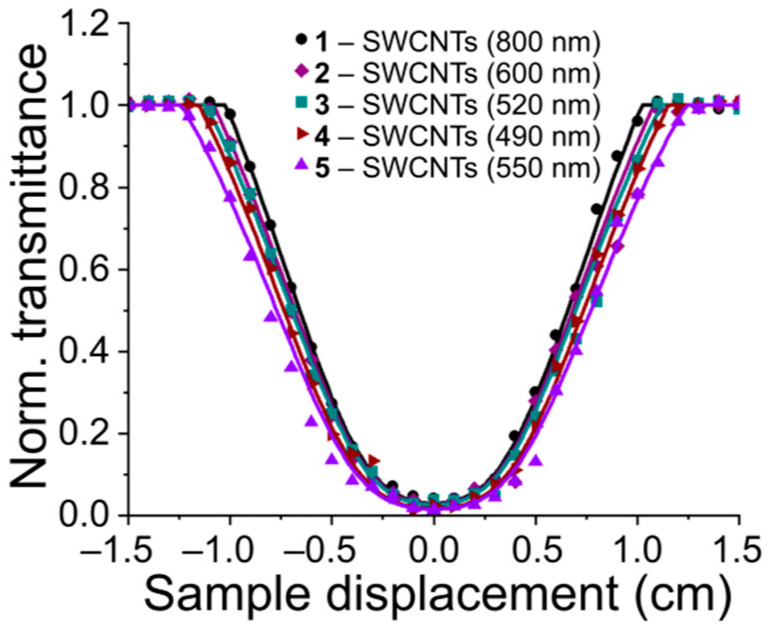
Open-aperture Z-scan results of Col/BSA/CS/Eosin Y/SWCNTs/rGO dispersions containing nanotubes with different hydrodynamic radii.

**Figure 4:**
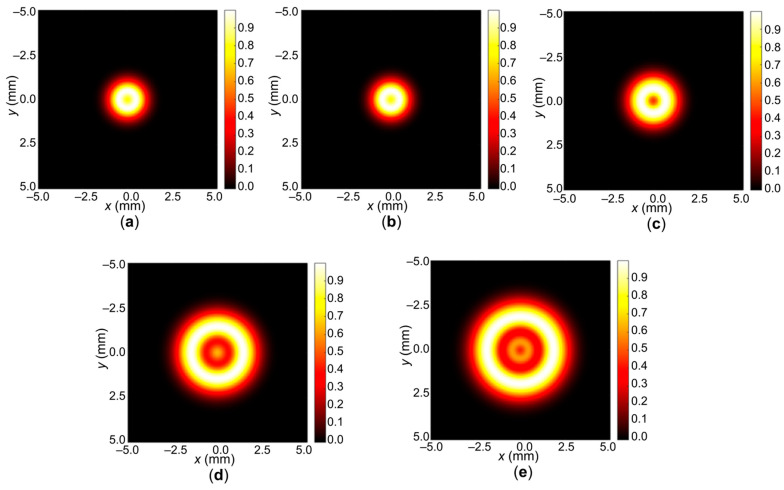
Results of modeling the diffraction ring patterns observed during spatial self-phase modulation for five compositions of photocurable media Col/BSA/CS/Eosin Y/SWCNTs/rGO: (**a**) **1**—SWCNTs (800 nm); (**b**) **2**—SWCNTs (600 nm); (**c**) **3**—SWCNTs (520 nm); (**d**) **4**—SWCNTs (490 nm); (**e**) **5**—SWCNTs (550 nm).

The cross-section of the nonlinear absorption coefficient σ was obtained using the open-aperture Z-scan method [24,42] (Figure 3) for five photocurable media. A decrease in the threshold exposure of laser radiation was found when nanotubes with a smaller hydrodynamic radius were used in the dispersion composition. For all samples, the nonlinear refractive index was determined taking into account the number of rings observed in the diffraction pattern. Figure 4 shows the results of the corresponding modeling.

The results of the diffraction patterns analysis revealed a strong influence of nonlinear refraction. The action of laser radiation on dispersions leads to a local gradient change in the refractive index n at the irradiation point in accordance with Equation (1):

(1) $$n=n_{0}+n_{n}\cdot I$$

where *n*_0_ is linear refractive index, *n*_n_ is nonlinear refractive index, and *I* is intensity of the incident laser radiation. A change in the refractive index under the influence of laser radiation results in a change in the phase of the wave front φ in accordance with Equation (2):

(2) $$\varphi =\frac{2\pi }{\lambda }\left(\int_{0}^{d}ndz-\int_{0}^{d}n_{0}dz\right)$$

where *d* is sample thickness. Such phase shift causes propagation velocity modulation for different sections of the laser beam and, as a consequence, a change in the spatial beam profile and the observation of a stable diffraction pattern. The method based on the Fresnel–Kirchhoff diffraction integral used to reproduce the diffraction ring pattern is described in detail in [43]. The modeling used values of linear and nonlinear refractive indexes, presented in Table 2.

**Figure 5:**
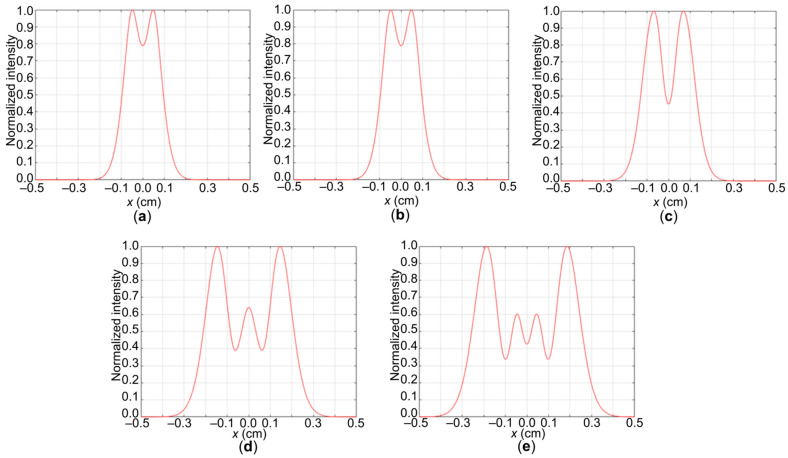
Projections along the *x*-axis of the diffraction pattern under self-phase modulation for five Col/BSA/CS/Eosin Y/SWCNTs/rGO dispersions: (**a**) **1**—SWCNTs (800 nm); (**b**) **2**—SWCNTs (600 nm); (**c**) **3**—SWCNTs (520 nm); (**d**) **4**—SWCNTs (490 nm); (**e**) **5**—SWCNTs (550 nm).

In the case of sample **1** (Figure 4a), the smallest spot diameter is observed, which approximately coincides with sample **2** (Figure 4b). Further, for sample **3**, the formation of a diffraction pattern with the formation of phase rings becomes noticeable (Figure 4c). In the case of samples **4** (Figure 4d) and **5** (Figure 4e), the diameter increases, and the rings themselves become more noticeable. For simplicity of analysis, projections of the normalized intensity along the *x*-axis are shown. In the case of samples **1** (Figure 5a) and **2** (Figure 5b), the presence of one ring is already visible. For sample **3** (Figure 5c), the difference in the normalized intensity increases and the ring becomes distinguishable. For samples **4** (Figure 5d) and **5** (Figure 5e), two rings are observed, which are better distinguishable for sample 5.

The influence of various factors on the subsequent process of hydrogel photopolymerization was analyzed using optical parameters, the values of which are presented in Table 2. When using nanotubes with the largest characteristic hydrodynamic radius in the composition of sample **1**, they exhibited a smaller nonlinear absorption cross-section σ = 750 GM and the highest threshold exposure value *F*_x_ = 0.09 J/cm^2^, while the refractive index was the lowest *n*_n_ = 0.26 cm/GW. In the case of samples **2**–**4**, some increase in nonlinear absorption values was observed due to an increase in scattering capacity, which was confirmed by the results of refractive index calculations, which varied from 0.26 cm^2^/GW to 0.78 cm^2^/GW. A trend toward a decrease in the laser exposure threshold from sample **1** to sample **5**, sufficient for nonlinear effects to manifest, was also detected. This is explained by scattering, which accelerates the increase in the material’s attenuation capacity. For sample 5, which contains SWCNTs with a characteristic hydrodynamic radius of 550 nm, this trend also persists, but the nonlinear absorption coefficient has actually decreased.

### 2.4. Specific Electrical Conductivity of Hydrogel

Hydrogel samples in the form of 5 × 5 mm^2^ squares were prepared for measurements of specific electrical conductivity using a four-probe station using the van der Pauw method at two temperatures: room temperature (25 °C) and intraorganismal temperature (37 °C) (Table 3). As a result, it was possible to improve this characteristic by more than three times compared to the value previously obtained in our previous work [14]. The highest electrical conductivity was obtained in the case of sample **4**, which used SWCNTs with a characteristic hydrodynamic radius of 490 nm. This result correlates well with the results of the conducted studies of the optical characteristics. It was with this composition of the Col/BSA/CS/Eosin Y/SWCNTs/rGO hydrogel that the lowest normalized transmittance was observed under the influence of laser pulses, after 80 s of irradiation (Figure 2d), and the largest nonlinear absorption cross-section (Table 2). This result demonstrates the importance of controlling ultrasonic treatment, which can improve properties to a certain extent. However, excessively long treatment can lead not only to bundle separation but also to their shortening, which can impair properties. For nanotubes in hydrogel **4**, treatment was performed for 80 min (Table 1).

**Figure 6:**
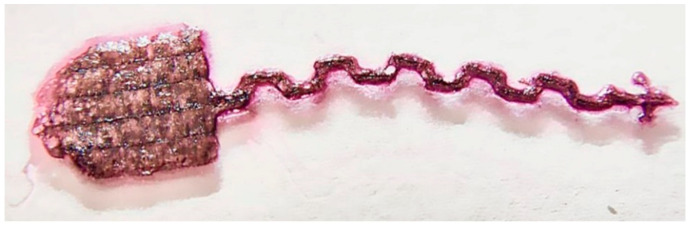
Complex structure made of Col/BSA/CS/Eosin Y/SWCNTs (490 nm)/rGO hydrogel (sample **4**).

Maintaining a temperature of 28 °C allowed not only for uniform polymerization, but also for the creation of complex structures (Figure 6), which had not previously been possible to create.

### 2.5. Hydrogel Degradation

**Figure 7:**
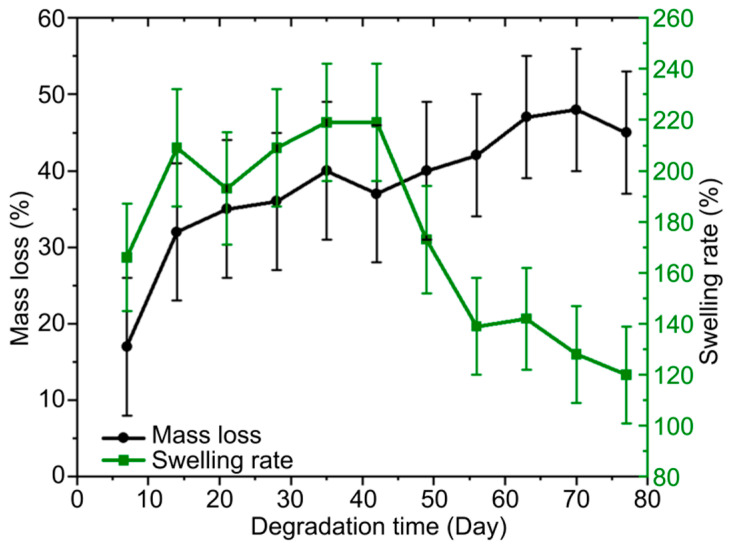
Mass loss and swelling rate of Col/BSA/CS/Eosin Y/SWCNTs (490 nm)/rGO hydrogel (sample 4) over an 80-day observation period.

Further studies were conducted with hydrogel **4**, which demonstrated the best electrical conductivity. The average mass loss of hydrogel **4** over 80 days was 45% (Figure 7). The initial swelling of the samples averaged 170% in isotonic solution. After 80 days of hydrolysis, the swelling rate decreased to 120%.

These results confirm the hydrogel’s ability to retain moisture and also its viscoelastic properties, which are important for ensuring tight contact between the neurostimulator electrodes and the moving nerves.

### 2.6. Biocompatibility

**Figure 8:**
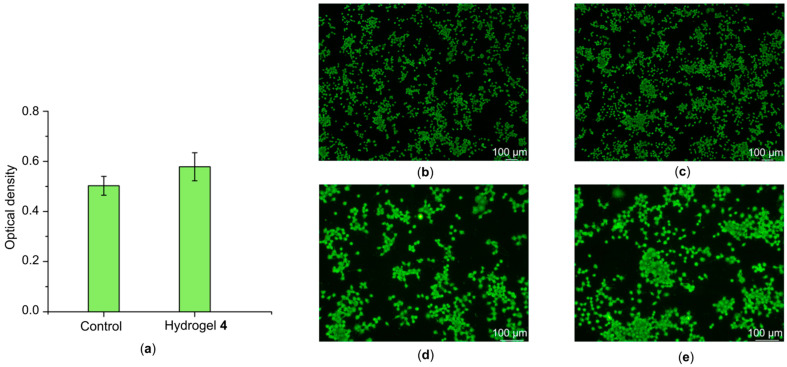
Biocompatibility study results after 72 h of Neuro-2A cell cultivation: (**a**) MTT assay; (**b**) fluorescent microscopy for control and (**c**) hydrogel Col/BSA/CS/Eosin Y/SWCNTs (490 nm)/rGO **4**; (**d**) fluorescent microscopy with a 2× increase for control and (**e**) hydrogel Col/BSA/CS/Eosin Y/SWCNTs (490 nm)/rGO **4**.

Biocompatibility was assessed using the Neuro 2A mouse neuroblastoma cell line, including a quantitative analysis of cell viability (MTT test) and a qualitative assessment of their morphology using fluorescence microscopy (Figure 8).

According to the MTT assay, incubation of cells for 72 h with hydrogel sample **4**, which exhibited superior conductivity, resulted in a 16% increase in cell proliferation compared to the control group (cells cultured under standard conditions without the addition of hydrogel). This result demonstrates the absence of a cytotoxic effect from the sample and attests to its stimulating effect on the proliferative activity of nerve cells.

rGO and SWCNTs both consist of sp_2_ C-C networks, but 2D flat graphene sheets exhibit significantly stronger interactions with biomolecules than 1D tubular SWCNTs [44]. This is due to the narrow hollow core of SWCNTs, which limits the penetration of biomolecules into the SWCNTs from the biological environment. In contrast, biomolecules interact with both the top and bottom surfaces of the rGO sheet. Moreover, the rough and wrinkled surface texture of rGO enhances its ability to interact with the surrounding biological environment, thereby enhancing the binding of biomolecules on its surface. In the case of rGO, toxicity is dose-dependent in both humans and animals, with low and medium doses having no effect in mice [45]. However, in the case of rGO, toxicity is lower when compared with the same amount of SWCNTs [31].

Analysis of images obtained using fluorescence microscopy revealed no differences in cell morphology between the experimental and control samples: the cells retained their characteristic shape and formed typical neurite-like processes. The cells in the experimental sample were more evenly distributed across the surface, which suggests potential for the formation of a disrupted neuronal network.

In the case of the previously used composition [24], the cell count was within acceptable limits, as demonstrated by the current study results. In the case of hydrogel **4**, the cell count exceeded the control results by 16%, while it was possible to use a larger amount of carbon material. The obtained results are also consistent with data from a number of studies demonstrating the biocompatibility and neurostimulating potential of biopolymer-based composite materials [46,47]. The inclusion of carbon nanomaterials contributes to improved electrical conductivity, which may facilitate the transmission of electrical signals important for nerve cells.

Thus, the obtained data allow us to conclude that sample **4** is highly biocompatible and has a beneficial effect on the viability and proliferation of nerve cells. These results confirm the potential for further use of this material in biomedical applications related to the creation of materials that interact with nerve tissue.

## 3. Conclusions

The research results demonstrate the feasibility of forming a hydrogel with complex geometry by maintaining a temperature of 28 °C during photopolymerization. Previous attempts at formation failed due to the presence of unpolymerized regions. The study revealed the influence of two factors on specific electrical conductivity: the characteristic hydrodynamic radius of SWCNTs, which indicates the size of their bundles, and the formation of their hybrid structures with rGO. The best performance was achieved with sample **4** (Col/BSA/CS/Eosin Y/SWCNTs/rGO) containing SWCNTs (490 nm). This sample exhibited a specific electrical conductivity of 72 mS × cm^−1^, surpassing the previously obtained value of 20 mS × cm^−1^ [24].

The conductive hydrogel material developed for electrodes enhances the performance of bionic devices designed to replace or augment sensory neuron functions. This improvement is due to increased operational stability and sustained close contact during swelling. The combination of the hydrogel matrix and SWCNTs promotes ion permeation and creates a three-dimensional interface that facilitates efficient charge transfer. Incorporated rGO flakes further increase the effective contact area. Consequently, the structured rGO–SWCNT network within the hydrogel substantially increases the charge-transfer surface area, enabling the electrode to deliver a higher charge at a lower operating voltage. This characteristic is essential for interfacing with weak biosignals during neural stimulation. Future studies will examine the effects of the concentration of individual components in the aqueous photopolymerizable medium on the following properties: specific electrical conductivity, degradation rate, swelling ratio, biocompatibility, sedimentation stability of the medium, and photopolymerization reproducibility. These findings will guide the optimization process and establish allowable concentration ranges for each component.

Such hydrogels are regarded not only for enhancing the stability of platinum–iridium electrodes [8] but also for expanding the application potential of materials such as titanium alloys [48] and medical-grade steel [2].

## 4. Materials and Methods

### 4.1. Preparation of Carbon Components

TUBALL™ (Lödelange, Luxembourg) nanotubes were selected as the SWCNTs, with a product content of 99 wt%. The material contains less than 1 wt% of other carbon allotropes and metallic impurities. It has a diameter of 1.6 ± 0.4 nm, a length of over 5 µm, and a specific surface area of 1000 m^2^/g [49,50]. We used reduced graphene oxide (rGO) from Grafenox LLC (Chernogolovka, Russia) with a specific surface area of 650 m^2^/g and a bulk density of 4–5 mg/cm^3^ [39,51].

To reduce bundle sizes and achieve a distribution closer to individual single-walled carbon nanotubes (SWCNTs), we used a Sonicator Q700 immersion ultrasonic homogenizer (Qsonica, Newtown, CT, USA) with a 1/2-inch (1.27 cm) diameter probe. Processing lasted two hours at a frequency of 20 kHz and power of 210 W in a 50 mL container. Thermostatic cooling prevented boiling due to the high-power ultrasound. This procedure yielded six samples of dispersed media, the data of which are presented in Table 1. We assessed the characteristic hydrodynamic radius of the SWCNT and rGO dispersions using dynamic light scattering (DLS) with a Photocor Compact instrument (Photocor, Moscow, Russia). This instrument determines particle sizes ranging from 0.5 nm to 10 µm. Each sample underwent five measurements, with a distinct color assigned to each set of measurements (Figure 1).

The procedure yielded one aqueous rGO dispersion (210 nm) after *t*_u_ = 80 min and five types of SWCNT dispersions following different ultrasonic treatment durations (*t*_u_): SWCNTs (800 nm) at *t*_u_ = 20 min, SWCNTs (600 nm) at *t*_u_ = 40 min, SWCNTs (520 nm) at *t*_u_ = 60 min, SWCNTs (490 nm) at *t*_u_ = 80 min, and SWCNTs (550 nm) at *t*_u_ = 100 min. For rGO, no significant changes in hydrodynamic radius occurred beyond *t*_u_ = 80 min of treatment; therefore, further processing was halted to prevent potential reduction in the lateral dimensions of the rGO flakes themselves.

### 4.2. Preparation of Photopolymerizable Media for Hydrogel Fabrication

**Figure 9:**
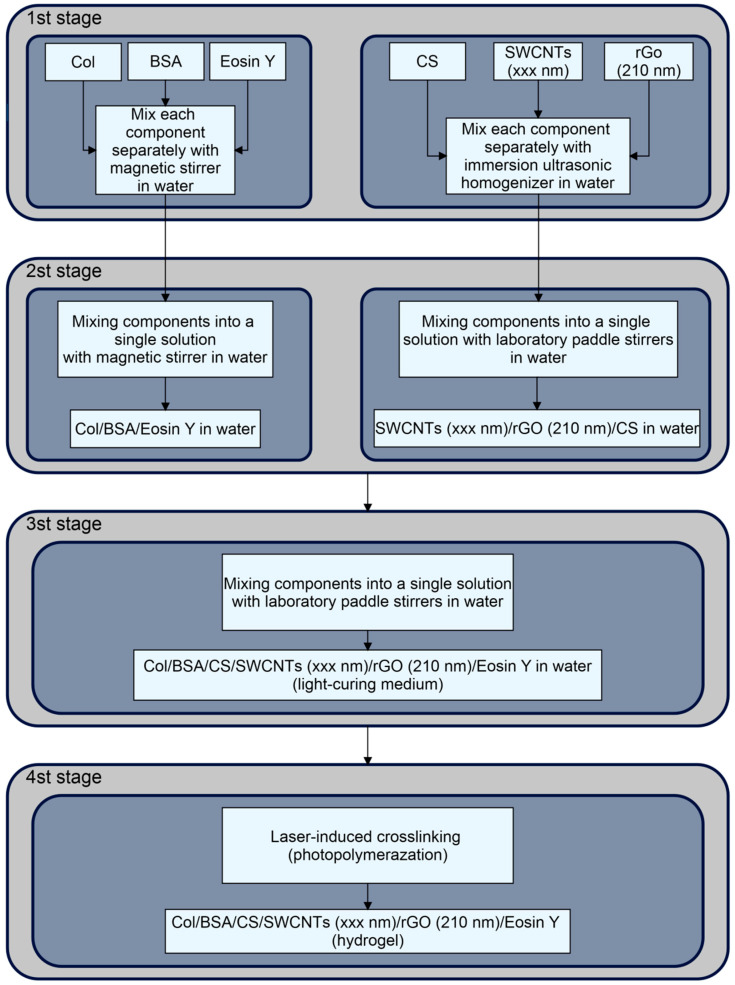
Preparation scheme for the Col/BSA/CS/Eosin Y/SWCNTs/rGO photopolymerizable medium. The arrows show the sequence of adding components and their subsequent processing.

The preparation of a medium suitable for use as a negative photoresist was carried out in three stages (Figure 9). The components included CS (Bioprogress LLC, Losino-Petrovsky, Russia), Col (MacMedi LLC, Moscow, Russia), and BSA (BioClot, Aidenbach, Germany). This procedure resulted in a uniform distribution of components throughout the entire volume.

In the first stage, aqueous solutions of eosin Y (Agat-Med, Moscow, Russia), BSA, and Col were prepared using an Elmi MS-01 magnetic stirrer (ELMI, Riga, Latvia). Concurrently, dispersions containing the carbon components (described in Section 4.1) and CS were prepared via ultrasonic treatment, as these components could not be adequately mixed using a magnetic stirrer.

The third stage involved mixing the samples obtained in the second stage together using a paddle stirrer. This process yielded five samples of photopolymerizable media (negative photoresists), each containing Col 25 mg/mL, BSA 50 mg/mL, CS 100 mg/mL, Eosin Y 1 mg/mL, rGO 0.3 mg/mL, and SWCNTs 0.3 mg/mL. The SWCNTs differed in their ultrasonic treatment duration (the hydrodynamic radius R of the particles with the greatest contribution is indicated in parentheses): **1**—SWCNTs (800 nm); **2**—SWCNTs (600 nm); **3**—SWCNTs (520 nm); **4**—SWCNTs (490 nm); **5**—SWCNTs (550 nm).

The fourth stage indicated in the scheme pertains to the direct formation of the hydrogel from the photopolymerizable medium. The photopolymerization process is described in more detail in Section 4.4.

### 4.3. Optical Studies and Temperature Control

**Figure 10:**
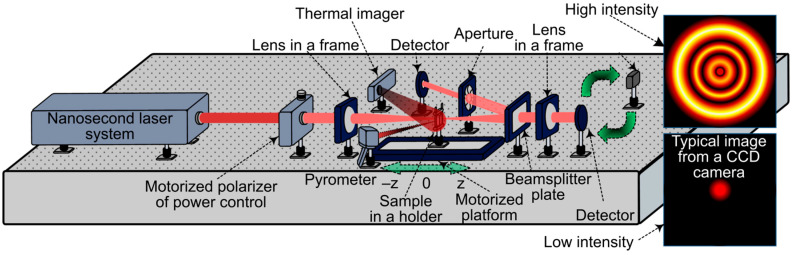
Schematic diagram of the experimental setup.

An HTTP MARK MOPA laser system (KB “Bulat”, Zelenograd, Russia) served as the radiation source, operating in a mode with a pulse duration of τ_n_ = 120 ns, a wavelength of λ = 1070 nm, and a pulse repetition frequency of ν = 15 kHz (Figure 10). A motorized polarizer provided smooth power adjustment. Following this, the radiation passed through a lens to achieve sufficient intensity for the manifestation of nonlinear effects. The sample was placed in a holder mounted on an N31.100E motorized stage (CoreMorrow Ltd., Harbin, China), controlled by an E71.D4E-H controller (CoreMorrow Ltd., Harbin, China) for movement along the beam propagation direction towards a second lens. Behind these optical components, a beamsplitter divided the beam into two paths. In one path, an aperture was placed before a power sensor; in the other, the beam directly illuminated a G5F-GT-10 power sensor (ShenZhen CaiHuang Thermoelectricity Technology Co. Ltd., Shenzhen, China). This configuration enabled Z-scan measurements and optical transmittance determination.

To determine the nonlinear absorption cross-section σ and the threshold energy fluence *F*_x_, the solution of the radiative transfer equation (RTE) was employed [24]. In this case, the normalized transmittance *T*_norm_ at constant incident power *P*_0_ depends on the sample displacement z relative to the lens focus, which relates to the change in energy fluence during focusing. For open-aperture Z-scanning, we use the previously derived [24] Equation (3):

(3) $$T_{norm}=\exp\left(\frac{d\sigma N_{A}C\cdot 10^{-3}}{\tau E_{\mathrm{ph}}\sqrt{\pi }}\left(F_{x}-\frac{2P_{0}z_{0}}{\nu \pi w_{0}^{2}\left(z_{0}^{2}+z^{2}\right)}\right)\right)$$

*d*—optical path length; *N*_A_—Avogadro constant; *C*—the molar concentration per liter; *τ*—pulse duration; *E*_ph_—the energy of the absorbed photon; z_0_—Rayleigh length; η—Heaviside function, which has only two values, 0 and 1; *w*_0_—beam radius at focus.

**Figure 11:**
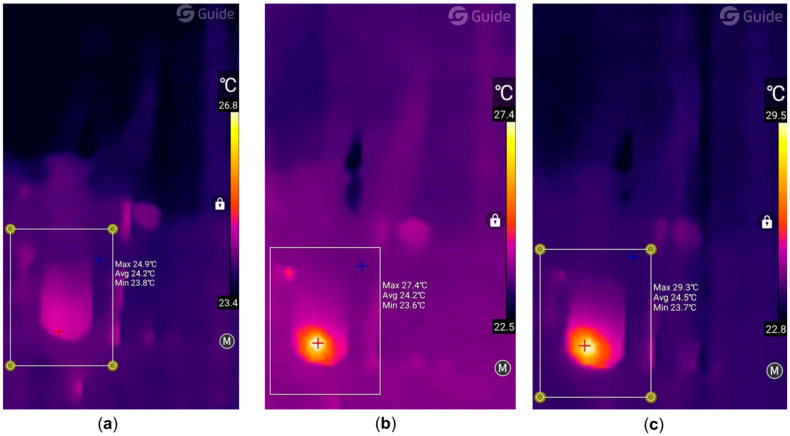
Typical images obtained by a thermal imager for sample **4** (Col/BSA/CS/Eosin Y/SWCNTs/rGO) with 490 nm SWCNTs: (**a**) initial moment, (**b**) photopolymerization, and (**c**) sample formation.

Temperature control was performed using a GM320 pyrometer (Benetech, Inc., Montgomery, IL, USA) and an IR USB Camera Module thermal imager (Wuhan Guide Sensmart, Ltd., Wuhan, China), which simplified the selection of the temperature recording location according to the method we used earlier [37]. Figure 11 shows typical thermal imager data during laser exposure.

**Figure 12:**
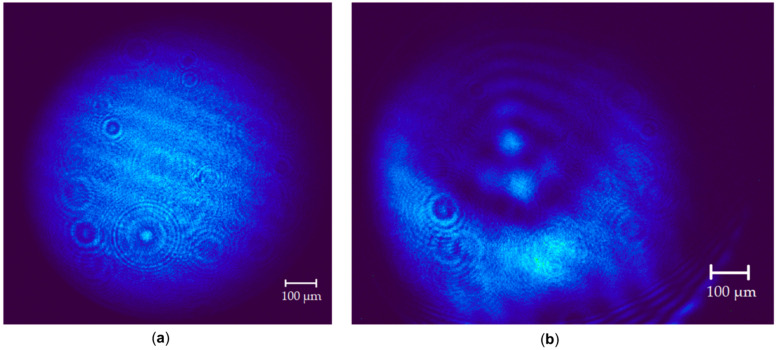
Diffraction rings pattern for sample **5** (Col/BSA/CS/Eosin Y/SWCNTs/rGO) with SWCNTs (550 nm): (**a**) at the initial moment; (**b**) under laser irradiation.

An SP920s laser profilometer (Ophir Optronics Ltd., Jerusalem, Israel), equipped with BeamGage Standard software (version 6.19), was used to determine the number of observable rings quantitatively. Figure 12 shows the data for sample 5, which contains SWCNTs (550 nm), and has two visible rings.

The number of diffraction rings, *N*, was determined based on data from a laser profilometer [24,52,53] with the sample in a fixed position. The nonlinear refractive index (*n_nr_*) was calculated using Equation (4):

(4) $$n_{nr}=\exp\left(\frac{\lambda \alpha N_{A}\nu w^{2}\tau \pi \sqrt{\pi }}{4n_{0}\left(1-\exp\left(-\alpha d\right)\right)P_{0}}\right)$$

*n*_0_—linear refractive index; α—linear absorption coefficient.

### 4.4. Studies of Electrical Conductivity and Hydrogel Formation

**Figure 13:**
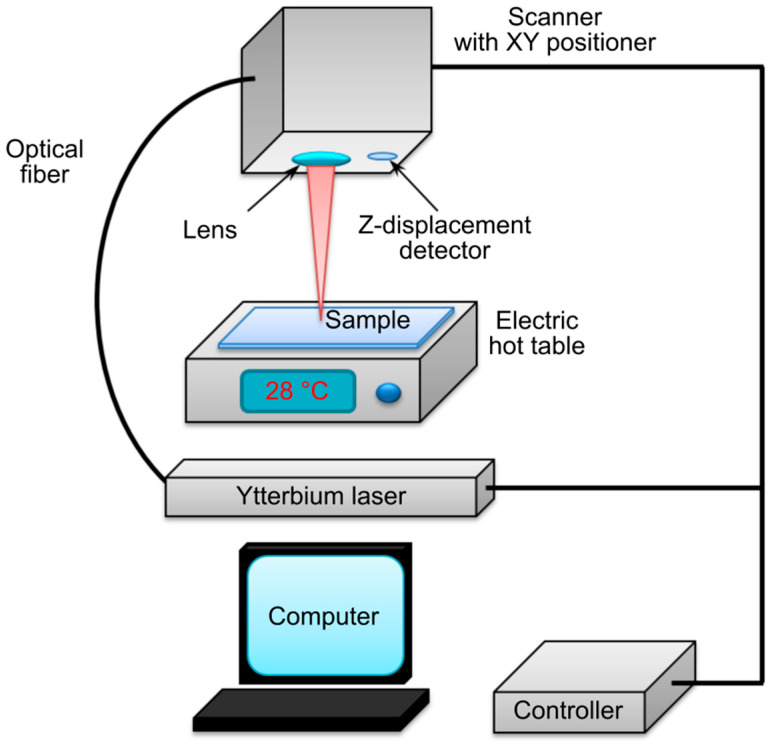
Schematic of the setup for photopolymerizing the Col/BSA/CS/Eosin Y/SWCNTs (490 nm)/rGO hydrogel.

To evaluate electrical conductivity, hydrogel samples were fabricated in a square shape measuring 5 × 5 mm^2^. Specific electrical conductivity was determined using the four-probe van der Pauw method (Suzhou Jingge Electronics Co., Ltd., Suzhou, China) at room temperature (25 °C) and a temperature close to the intraorganismic environment (37 °C). Photopolymerization was carried out using a fiber laser equipped with a scanning system (Figure 13), delivering pulses with a duration of 100 ns, a repetition rate of 30 kHz, and a wavelength of 1070 nm. The radius of the laser spot at the objective focus was 19 µm. The laser trajectory was computer-controlled. The laser power was set above the threshold exposure determined from the temperature-versus-time studies. This setting was necessary to achieve photopolymerization at a scanning speed of 240 mm/s with a single pulse energy of ~100 µJ. In the presence of Col, CS, and BSA molecules containing cationic groups, ionic interactions with the anions of Eosin Y are induced, accompanied by hydrogen bond formation. SWCNTs and rGO further connect via weak van der Waals forces, acting as secondary stabilization mechanisms, as confirmed by spectroscopic analysis [24]. Based on the analysis of the temperature dependence on irradiation time, a working temperature of 28 °C was established and maintained by a heating stage.

### 4.5. Degradation Studies

A study of the degradation of hydrogel **4** (erosion, swelling, passive hydrolysis) was conducted, since this sample demonstrated the best specific electrical conductivity. To simulate the intraorganic environment, the samples were kept in an isotonic saline solution (0.9% NaCl) (Spaz Farm LLC., Saratov, Russia) at a temperature of 37 °C. The degradation rate was assessed over 80 days with control measurements every 7 days. The samples were kept in 5 mL of solution at a constant temperature of 37 °C. The repeated degree of swelling was estimated using Equation (5) [54,55]:

(5) $$S_{t}=\frac{m_{wt}-m_{t}}{m_{o}}\times 100\%,$$

where *S*_t_ is the degree of re-swelling after *t* days of degradation, *m*_wt_ is the mass of the wet swollen sample after *t* days, *m*_0_ is the initial mass of the dry sample before decomposition, and *m*_t_ is its mass after *t* days.

The mass loss was estimated using Equation (6) [54,55]:

(6) $$M_{t}=\frac{m_{o}-m_{t}}{m_{o}}\times 100\%,$$

where *M*_t_ is the mass loss after *t* days of degradation.

### 4.6. Biocompatibility Studies

The Neuro-2A mouse glioblastoma cell line, obtained at the N.F. Gamaleya National Research Center for Epidemiology and Microbiology, was used for biocompatibility studies. Cells were cultured in Dulbecco’s modified eagle medium (DMEM) (BioloT LLC., Saint Petersburg, Russia) supplemented with 10% bovine calf serum (BioloT LLC., Saint Petersburg, Russia). Cell seeding doses were determined using a Scepter Millipore cell counter (Merck KGaA, Darmstadt, Germany). Samples were placed at the bottom of wells in a culture plate, and a cell suspension at a seeding dose of 2.3 × 10^5^ cells/mL was added to each sample. The plates with samples were kept in a CO_2_ incubator for 72 h after cell seeding. At the end of incubation, the MTT test was performed according to the standard method [56,57], and the optical density was read automatically using an Immunochem-2100 microplate photocalorimeter (High Technology Inc., North Attleboro, MA, USA). To assess cellular morphology at the end of incubation, the cells on the samples were stained with Hoechst 33,342 dye and examined using an Olympus IX73 fluorescence microscope (Olympus Corporation, Tokyo, Japan).

## Figures and Tables

**Table 1:** DLS results for dispersions of SWCNTs and rGO.

Ultrasonic Treatment Time, min	Ultrasound Power, W	Type of Nanoparticles	Hydrodynamic Radius *R*, nm	Standard Deviation, nm	Contribution, %
20	210	Individual SWCNTs	195	35	18
		SWCNT Bundles	800	240	76
		SWCNT Bundles	670,000	180,000	6
40	210	Individual SWCNTs	65	4	5
		SWCNT Bundles	600	90	88
		SWCNT Bundles	800,000	120,000	7
60	210	Individual SWCNTs	60	4	2
		SWCNT Bundles	520	100	94
		SWCNT Bundles	790,000	130,000	4
80	210	Individual SWCNTs	40	5	2
		SWCNT Bundles	490	100	88
		SWCNT Bundles	180,000	110,000	10
100	210	Individual SWCNTs	70	4	3
		SWCNT Bundles	550	70	90
		SWCNT Bundles	860,000	96,000	7
80	210	Individual rGO	45	5	9
		Individual rGO	210	50	90
		rGO agglomerates	920,000	65,000	1

**Table 2:** Properties of photocurable media Col/BSA/CS/Eosin Y/SWCNTs/rGO.

Photocurable Medium (Negative Photoresist)	Hydrodynamic Radius of SWCNTs *R*, nm	Linear Absorption Coefficient α, cm^−1^	Nonlinear Absorption Cross-Section σ, GM	Threshold Laser Exposure *F*_x_, J/cm^2^	Linear Refractive Index *n*_0_	Nonlinear Refractive Index *n*_n_, cm^2^/GW
**1**	800 ± 200	40 ± 3	750 ± 50	0.09 ± 0.01	1.353 ± 0.001	0.26 ± 0.02
**2**	600 ± 90	37 ± 3	770 ± 50	0.08 ± 0.01	1.356 ± 0.001	0.26 ± 0.02
**3**	520 ± 90	40 ± 3	760 ± 50	0.08 ± 0.01	1.359 ± 0.001	0.42 ± 0.02
**4**	490 ± 40	40 ± 3	850 ± 60	0.07 ± 0.01	1.361 ± 0.001	0.78 ± 0.03
**5**	550 ± 70	35 ± 3	830 ± 60	0.06 ± 0.01	1.363 ± 0.001	0.95 ± 0.03

**Table 3:** Electrical conductivity of the formed Col/BSA/CS/Eosin Y/SWCNTs/rGO hydrogel.

Hydrogel	Hydrodynamic Radius of SWCNTs *R*, nm	Specific Conductivity (25 °C), mS × cm^−1^	Specific Conductivity (37 °C), mS × cm^−1^
**1**	800 ± 200	28 ± 2	35 ± 2
**2**	600 ± 90	18 ± 2	22 ± 2
**3**	520 ± 90	32 ± 2	38 ± 2
**4**	490 ± 40	72 ± 6	86 ± 6
**5**	550 ± 70	26 ± 2	31 ± 2

## Data Availability

Data underlying the results presented in this paper are not publicly available at this time but may be obtained from the corresponding authors upon reasonable request.

## Abbreviations

The following abbreviations are used in this manuscript:

CNS	Central nervous system
DC	Direct current
SCS	Spinal cord stimulation
Col	Collagen
BSA	Bovine serum albumin
CS	Chitosan
SWCNTs	Single-wall carbon nanotubes
DLS	Dynamic light scattering
rGO	Reduced graphene oxide
Eosin Y	Eosin Yellow
MTT	Colorimetric assay for assessing cell metabolic activity
RTE	Radiative transfer equation
DMEM	Dulbecco’s modified eagle medium
